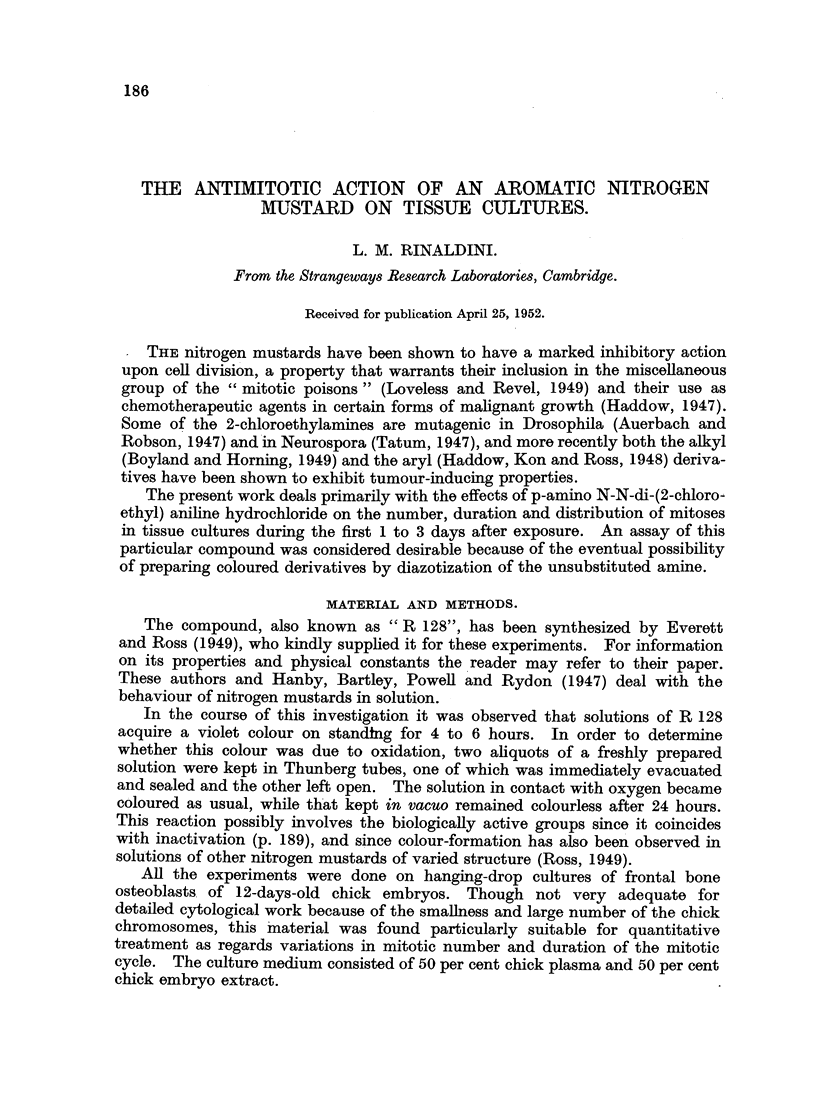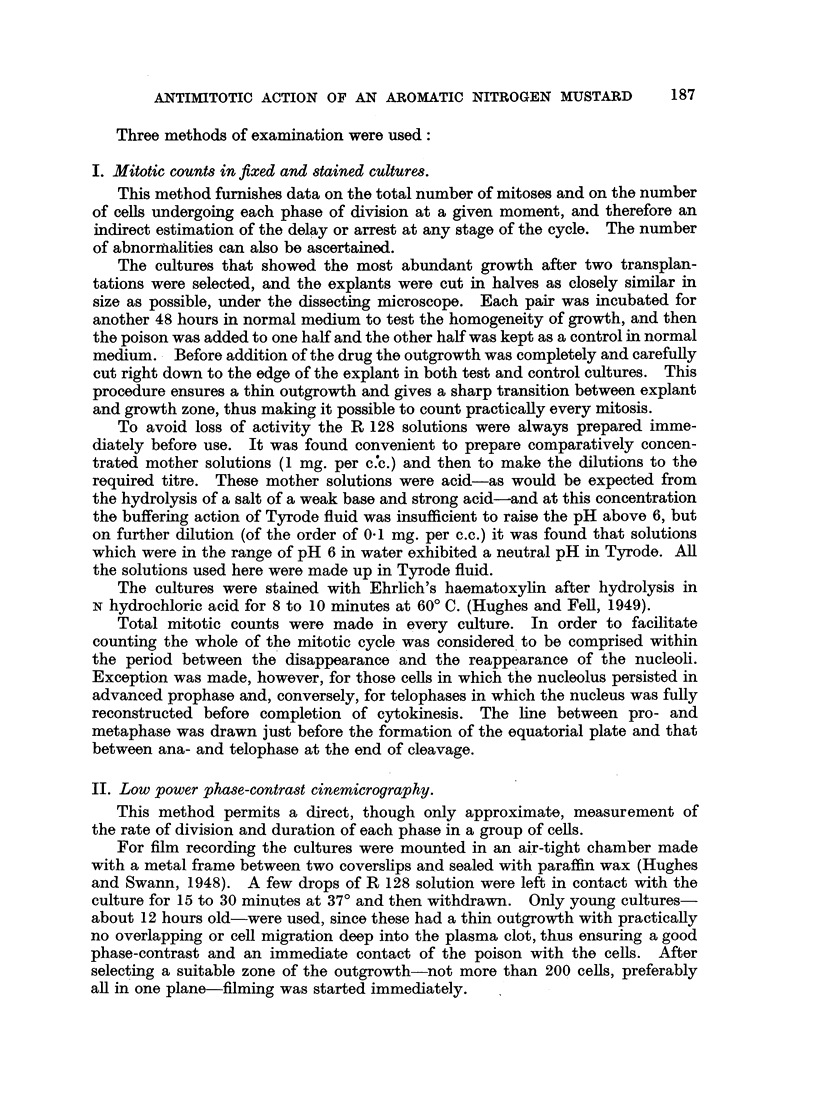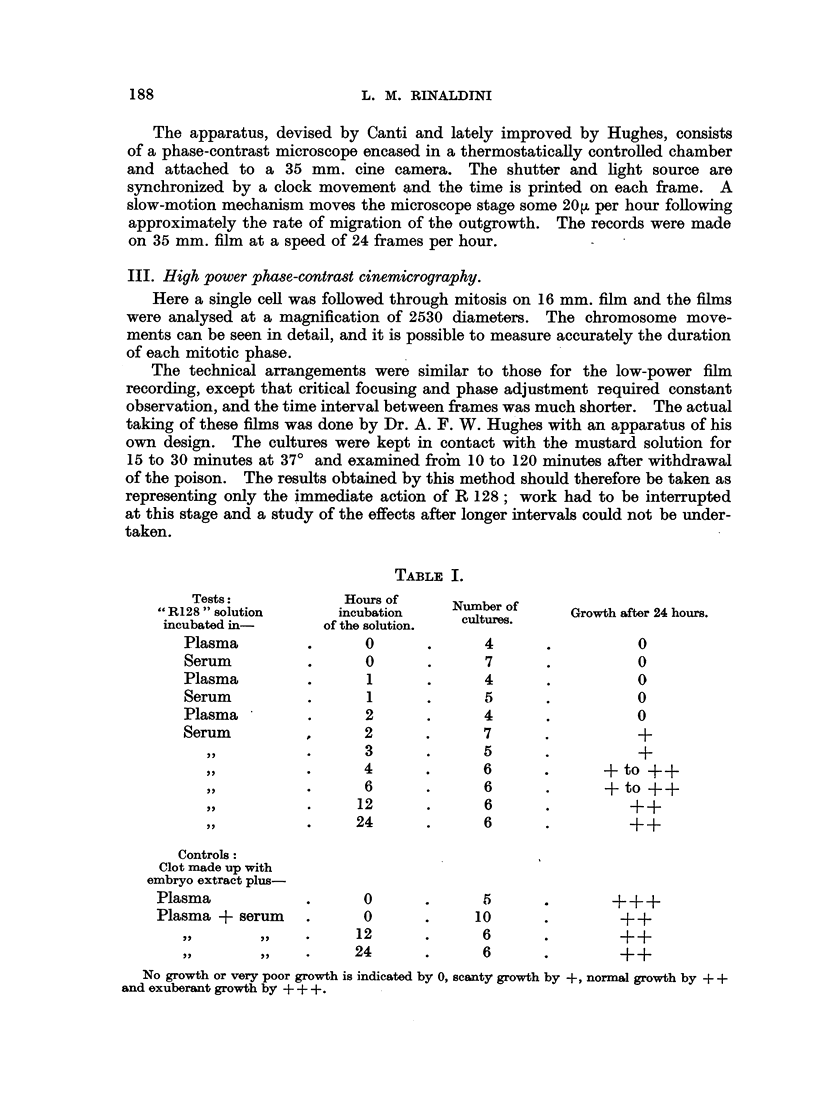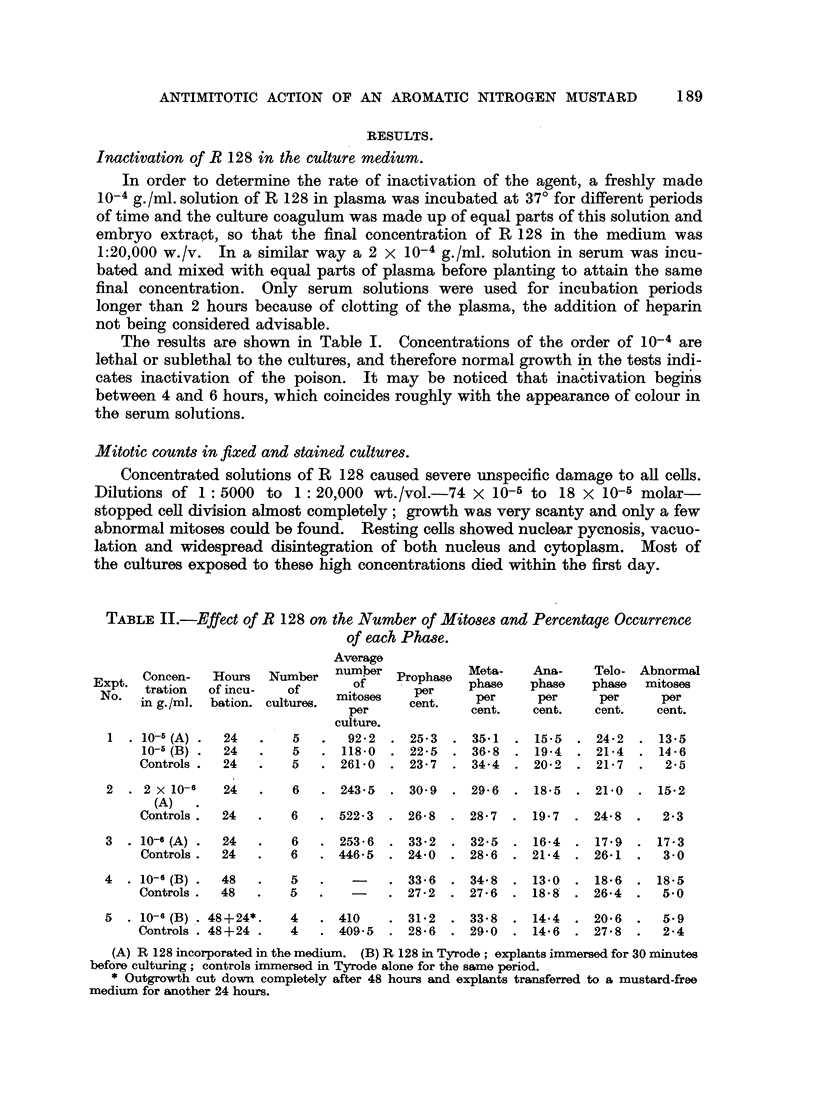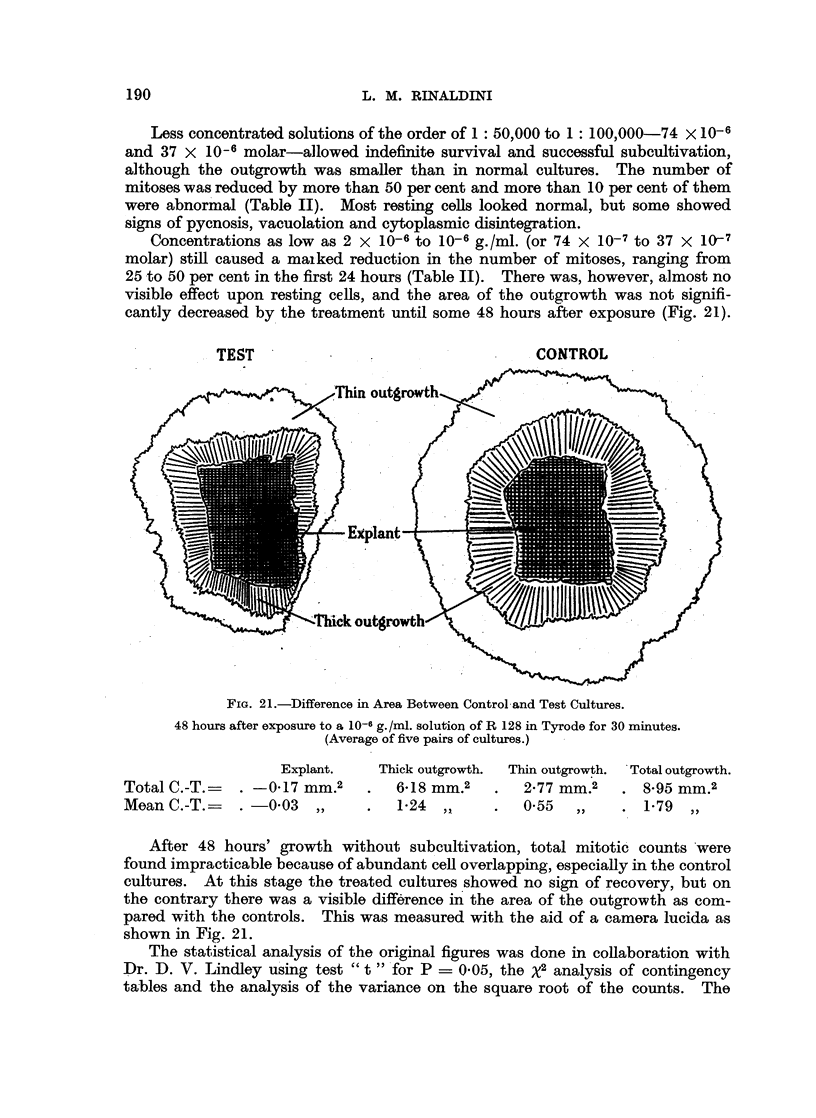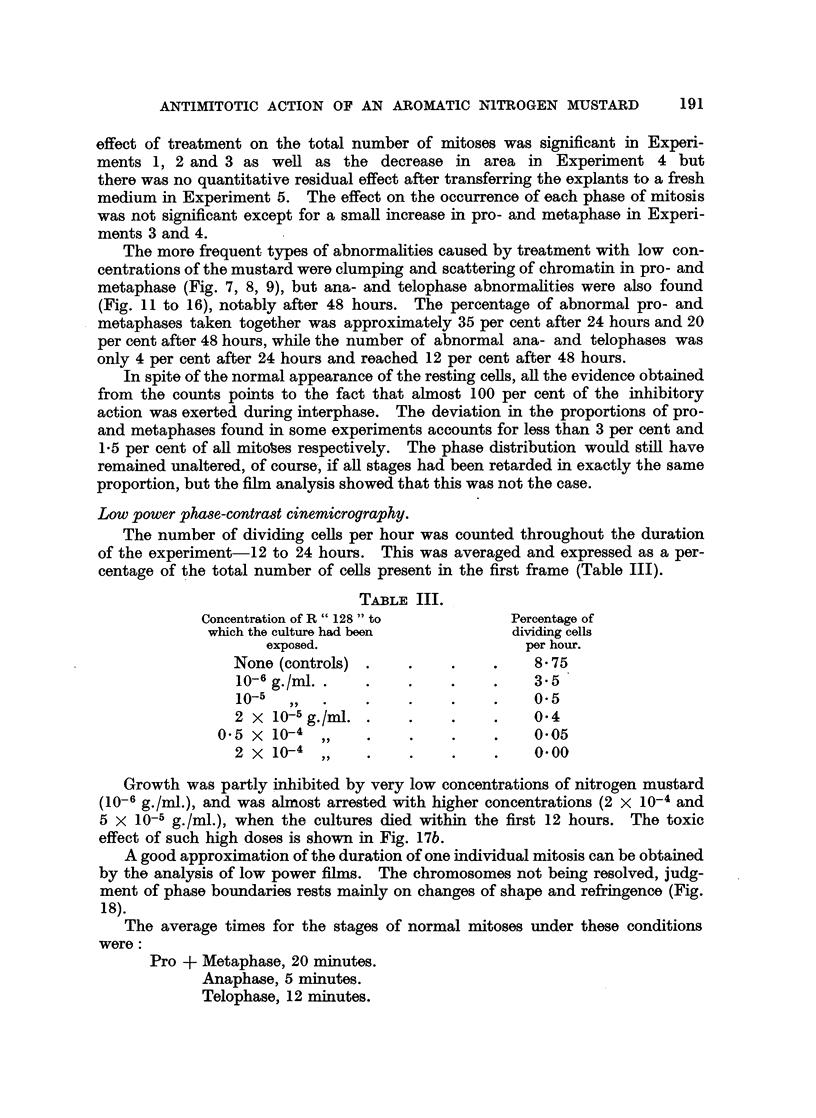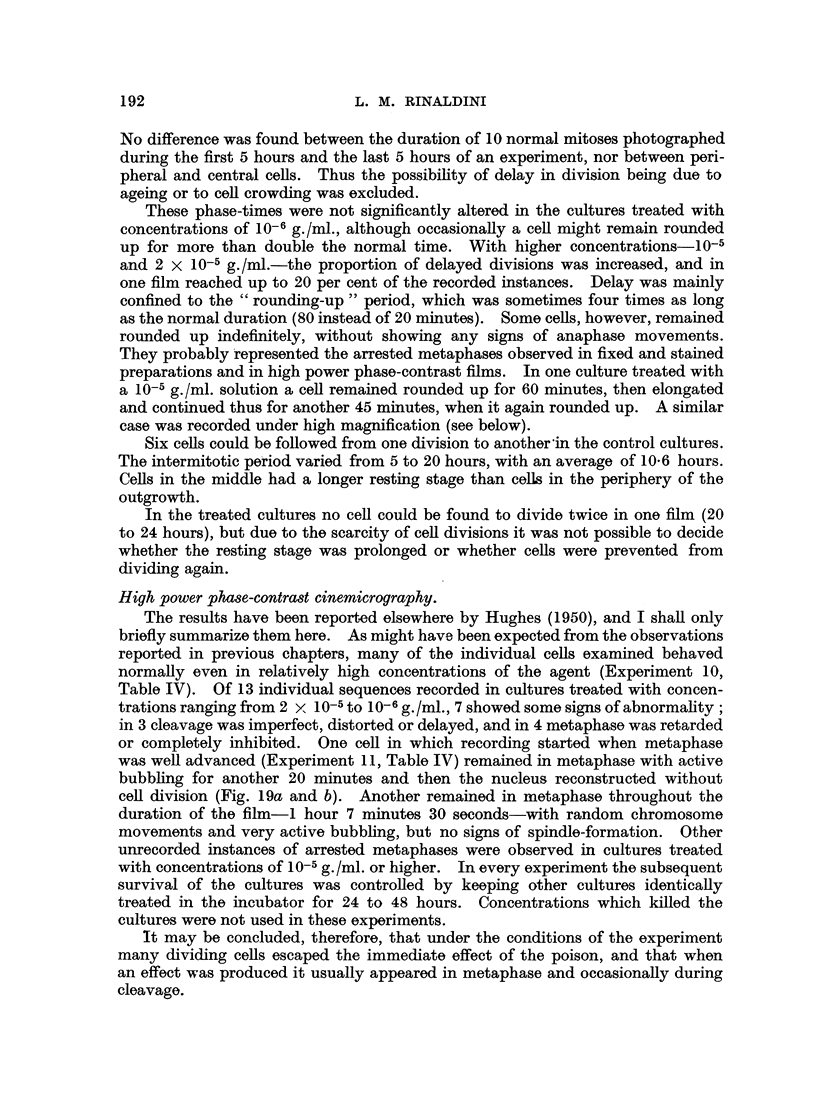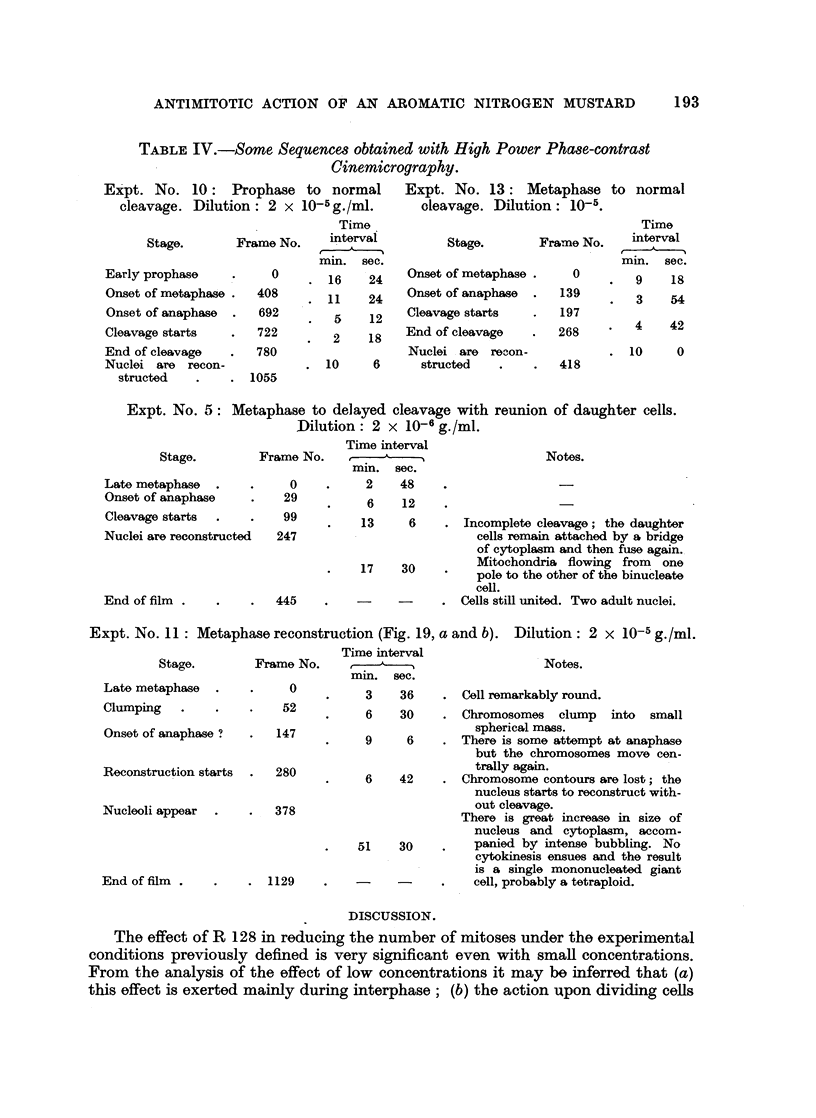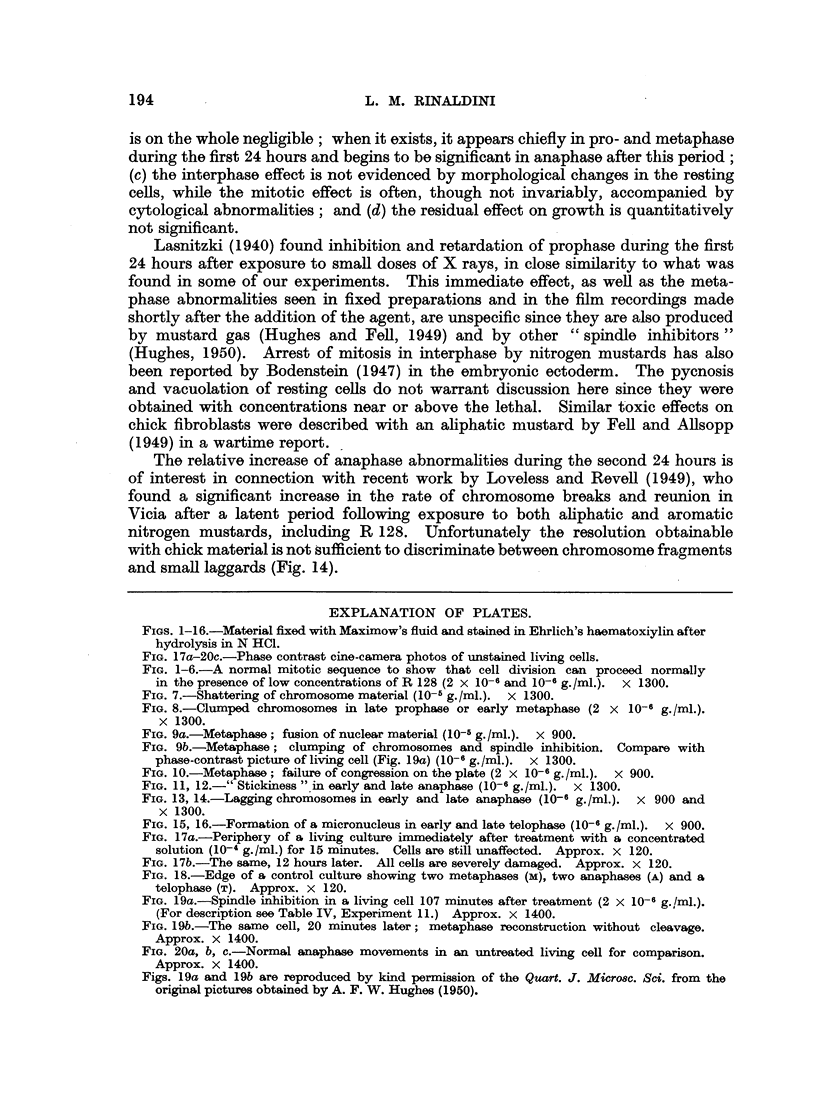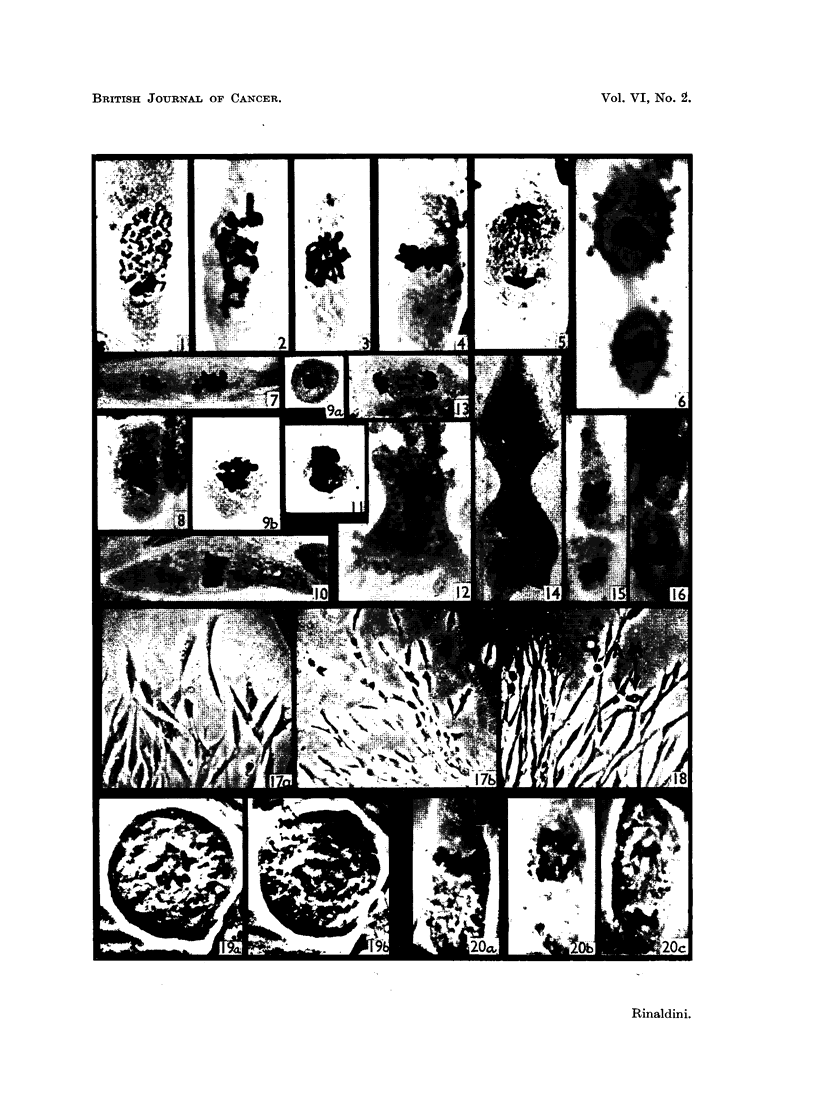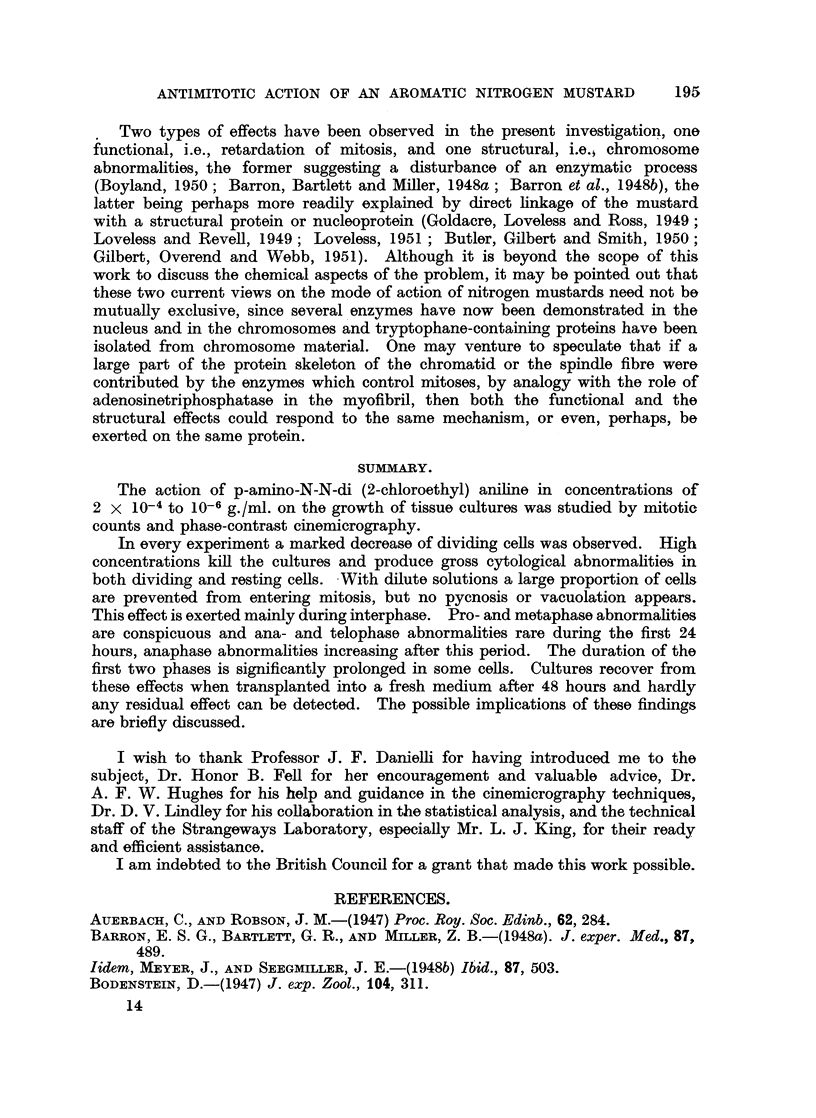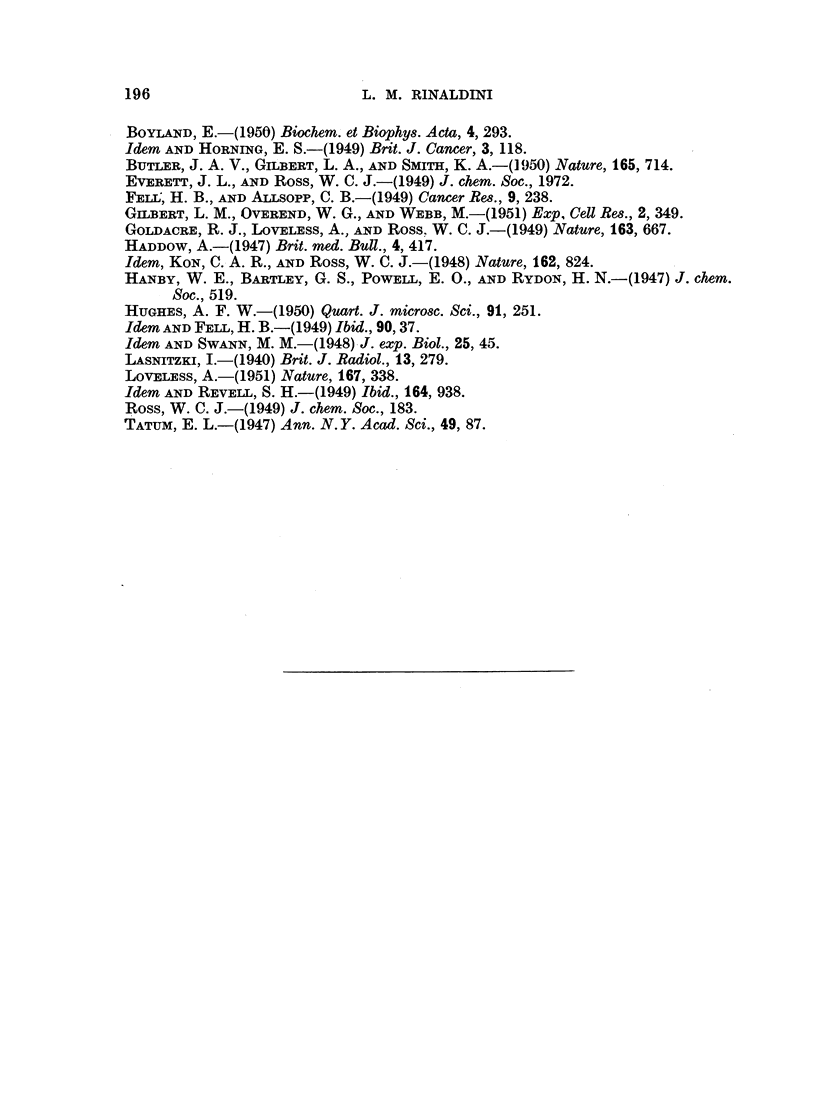# The Antimitotic Action of an Aromatic Nitrogen Mustard on Tissue Cultures

**DOI:** 10.1038/bjc.1952.21

**Published:** 1952-06

**Authors:** L. M. Rinaldini

## Abstract

**Images:**


### THE ANTIMITOTIC ACTION OF AN AROMATIC NITROGEN MUSTARD ON TISSUE CULTURES.

L. M. RINALDINI.

*From the Strangeways Research Laboratories, Cambridge.*

Received for publication April 25, 1952.

THE nitrogen mustards have been shown to have a marked inhibitory action upon cell division, a property that warrants their inclusion in the miscellaneous group of the " mitotic poisons " (Loveless and Revel, 1949) and their use as chemotherapeutic agents in certain forms of malignant growth (Haddow, 1947). Some of the 2-chloroethylamines are mutagenic in Drosophila (Auerbach and Robson, 1947) and in Neurospora (Tatum, 1947), and more recently both the alkyl (Boyland and Horning, 1949) and the aryl (Haddow, Kon and Ross, 1948) derivatives have been shown to exhibit tumour-inducing properties.

The present work deals primarily with the effects of p-amino N-N-di-(2-chloroethyl) aniline hydrochloride on the number, duration and distribution of mitoses in tissue cultures during the first 1 to 3 days after exposure. An assay of this particular compound was considered desirable because of the eventual possibility of preparing coloured derivatives by diazotization of the unsubstituted amine.

### MATERIAL AND METHODS.

The compound, also known as " R 128", has been synthesized by Everett and Ross (1949), who kindly supplied it for these experiments. For information on its properties and physical constants the reader may refer to their paper. These authors and Hanby, Bartley, Powell and Rydon (1947) deal with the behaviour of nitrogen mustards in solution.

In the course of this investigation it was observed that solutions of R 128 acquire a violet colour on standing for 4 to 6 hours. In order to determine whether this colour was due to oxidation, two aliquots of a freshly prepared solution were kept in Thunberg tubes, one of which was immediately evacuated and sealed and the other left open. The solution in contact with oxygen became coloured as usual, while that kept *in vacuo* remained colourless after 24 hours. This reaction possibly involves the biologically active groups since it coincides with inactivation (p. 189), and since colour-formation has also been observed in solutions of other nitrogen mustards of varied structure (Ross, 1949).

All the experiments were done on hanging-drop cultures of frontal bone osteoblasts of 12-days-old chick embryos. Though not very adequate for detailed cytological work because of the smallness and large number of the chick chromosomes, this material was found particularly suitable for quantitative treatment as regards variations in mitotic number and duration of the mitotic cycle. The culture medium consisted of 50 per cent chick plasma and 50 per cent chick embryo extract.

Three methods of examination were used :

I. *Mitotic counts in fixed and stained cultures.*

This method furnishes data on the total number of mitoses and on the number of cells undergoing each phase of division at a given moment, and therefore an indirect estimation of the delay or arrest at any stage of the cycle. The number of abnormalities can also be ascertained.

The cultures that showed the most abundant growth after two transplantations were selected, and the explants were cut in halves as closely similar in size as possible, under the dissecting microscope. Each pair was incubated for another 48 hours in normal medium to test the homogeneity of growth, and then the poison was added to one half and the other half was kept as a control in normal medium. Before addition of the drug the outgrowth was completely and carefully cut right down to the edge of the explant in both test and control cultures. This procedure ensures a thin outgrowth and gives a sharp transition between explant and growth zone, thus making it possible to count practically every mitosis.

To avoid loss of activity the R 128 solutions were always prepared immediately before use. It was found convenient to prepare comparatively concentrated mother solutions (1 mg. per c.c.) and then to make the dilutions to the required titre. These mother solutions were acid—as would be expected from the hydrolysis of a salt of a weak base and strong acid—and at this concentration the buffering action of Tyrode fluid was insufficient to raise the pH above 6, but on further dilution (of the order of 0·1 mg. per c.c.) it was found that solutions which were in the range of pH 6 in water exhibited a neutral pH in Tyrode. All the solutions used here were made up in Tyrode fluid.

The cultures were stained with Ehrlich's haematoxylin after hydrolysis in N hydrochloric acid for 8 to 10 minutes at 60° C. (Hughes and Fell, 1949).

Total mitotic counts were made in every culture. In order to facilitate counting the whole of the mitotic cycle was considered to be comprised within the period between the disappearance and the reappearance of the nucleoli. Exception was made, however, for those cells in which the nucleolus persisted in advanced prophase and, conversely, for telophases in which the nucleus was fully reconstructed before completion of cytokinesis. The line between pro- and metaphase was drawn just before the formation of the equatorial plate and that between ana- and telophase at the end of cleavage.

II. *Low power phase-contrast cinemicrography.*

This method permits a direct, though only approximate, measurement of the rate of division and duration of each phase in a group of cells.

For film recording the cultures were mounted in an air-tight chamber made with a metal frame between two coverslips and sealed with paraffin wax (Hughes and Swann, 1948). A few drops of R 128 solution were left in contact with the culture for 15 to 30 minutes at 37° and then withdrawn. Only young cultures—about 12 hours old—were used, since these had a thin outgrowth with practically no overlapping or cell migration deep into the plasma clot, thus ensuring a good phase-contrast and an immediate contact of the poison with the cells. After selecting a suitable zone of the outgrowth—not more than 200 cells, preferably all in one plane—filming was started immediately.

The apparatus, devised by Canti and lately improved by Hughes, consists of a phase-contrast microscope encased in a thermostatically controlled chamber and attached to a 35 mm. cine camera. The shutter and light source are synchronized by a clock movement and the time is printed on each frame. A slow-motion mechanism moves the microscope stage some 20$\mu$ per hour following approximately the rate of migration of the outgrowth. The records were made on 35 mm. film at a speed of 24 frames per hour.

III. *High power phase-contrast cinemicrography.*

Here a single cell was followed through mitosis on 16 mm. film and the films were analysed at a magnification of 2530 diameters. The chromosome movements can be seen in detail, and it is possible to measure accurately the duration of each mitotic phase.

The technical arrangements were similar to those for the low-power film recording, except that critical focusing and phase adjustment required constant observation, and the time interval between frames was much shorter. The actual taking of these films was done by Dr. A. F. W. Hughes with an apparatus of his own design. The cultures were kept in contact with the mustard solution for 15 to 30 minutes at 37° and examined from 10 to 120 minutes after withdrawal of the poison. The results obtained by this method should therefore be taken as representing only the immediate action of R 128 ; work had to be interrupted at this stage and a study of the effects after longer intervals could not be undertaken.

TABLE I.

| Tests: "R128" solution incubated in— | Hours of incubation of the solution. | Number of cultures. | Growth after 24 hours. |
|---|---|---|---|
| Plasma | 0 | 4 | 0 |
| Serum | 0 | 7 | 0 |
| Plasma | 1 | 4 | 0 |
| Serum | 1 | 5 | 0 |
| Plasma | 2 | 4 | 0 |
| Serum | 2 | 7 | + |
| ,, | 3 | 5 | + |
| ,, | 4 | 6 | + to ++ |
| ,, | 6 | 6 | + to ++ |
| ,, | 12 | 6 | ++ |
| ,, | 24 | 6 | ++ |
| Controls: Clot made up with embryo extract plus— | | | |
| Plasma | 0 | 5 | +++ |
| Plasma + serum | 0 | 10 | ++ |
| ,, ,, | 12 | 6 | ++ |
| ,, ,, | 24 | 6 | ++ |

No growth or very poor growth is indicated by 0, scanty growth by +, normal growth by ++ and exuberant growth by +++.

### RESULTS.

### *Inactivation of R 128 in the culture medium.*

In order to determine the rate of inactivation of the agent, a freshly made $10^{-4}$ g./ml. solution of R 128 in plasma was incubated at 37° for different periods of time and the culture coagulum was made up of equal parts of this solution and embryo extract, so that the final concentration of R 128 in the medium was 1:20,000 w./v. In a similar way a $2 \times 10^{-4}$ g./ml. solution in serum was incubated and mixed with equal parts of plasma before planting to attain the same final concentration. Only serum solutions were used for incubation periods longer than 2 hours because of clotting of the plasma, the addition of heparin not being considered advisable.

The results are shown in Table I. Concentrations of the order of $10^{-4}$ are lethal or sublethal to the cultures, and therefore normal growth in the tests indicates inactivation of the poison. It may be noticed that inactivation begins between 4 and 6 hours, which coincides roughly with the appearance of colour in the serum solutions.

### *Mitotic counts in fixed and stained cultures.*

Concentrated solutions of R 128 caused severe unspecific damage to all cells. Dilutions of 1 : 5000 to 1 : 20,000 wt./vol.—$74 \times 10^{-5}$ to $18 \times 10^{-5}$ molar—stopped cell division almost completely ; growth was very scanty and only a few abnormal mitoses could be found. Resting cells showed nuclear pycnosis, vacuolation and widespread disintegration of both nucleus and cytoplasm. Most of the cultures exposed to these high concentrations died within the first day.

TABLE II.—*Effect of R 128 on the Number of Mitoses and Percentage Occurrence of each Phase.*

| Expt. No. | Concentration in g./ml. | Hours of incubation. | Number of cultures. | Average number of mitoses per culture. | Prophase per cent. | Metaphase per cent. | Anaphase per cent. | Telophase per cent. | Abnormal mitoses per cent. |
|---|---|---|---|---|---|---|---|---|---|
| 1 | $10^{-5}$ (A) | 24 | 5 | 92·2 | 25·3 | 35·1 | 15·5 | 24·2 | 13·5 |
| | $10^{-5}$ (B) | 24 | 5 | 118·0 | 22·5 | 36·8 | 19·4 | 21·4 | 14·6 |
| | Controls | 24 | 5 | 261·0 | 23·7 | 34·4 | 20·2 | 21·7 | 2·5 |
| 2 | $2 \times 10^{-6}$ (A) | 24 | 6 | 243·5 | 30·9 | 29·6 | 18·5 | 21·0 | 15·2 |
| | Controls | 24 | 6 | 522·3 | 26·8 | 28·7 | 19·7 | 24·8 | 2·3 |
| 3 | $10^{-6}$ (A) | 24 | 6 | 253·6 | 33·2 | 32·5 | 16·4 | 17·9 | 17·3 |
| | Controls | 24 | 6 | 446·5 | 24·0 | 28·6 | 21·4 | 26·1 | 3·0 |
| 4 | $10^{-6}$ (B) | 48 | 5 | — | 33·6 | 34·8 | 13·0 | 18·6 | 18·5 |
| | Controls | 48 | 5 | — | 27·2 | 27·6 | 18·8 | 26·4 | 5·0 |
| 5 | $10^{-6}$ (B) | 48+24* | 4 | 410 | 31·2 | 33·8 | 14·4 | 20·6 | 5·9 |
| | Controls | 48+24 | 4 | 409·5 | 28·6 | 29·0 | 14·6 | 27·8 | 2·4 |

(A) R 128 incorporated in the medium. (B) R 128 in Tyrode ; explants immersed for 30 minutes before culturing ; controls immersed in Tyrode alone for the same period.

* Outgrowth cut down completely after 48 hours and explants transferred to a mustard-free medium for another 24 hours.

Less concentrated solutions of the order of 1 : 50,000 to 1 : 100,000—74 $\times 10^{-6}$ and 37 $\times$ $10^{-6}$ molar—allowed indefinite survival and successful subcultivation, although the outgrowth was smaller than in normal cultures. The number of mitoses was reduced by more than 50 per cent and more than 10 per cent of them were abnormal (Table II). Most resting cells looked normal, but some showed signs of pycnosis, vacuolation and cytoplasmic disintegration.

Concentrations as low as 2 $\times$ $10^{-6}$ to $10^{-6}$ g./ml. (or 74 $\times$ $10^{-7}$ to 37 $\times$ $10^{-7}$ molar) still caused a maıked reduction in the number of mitoses, ranging from 25 to 50 per cent in the first 24 hours (Table II). There was, however, almost no visible effect upon resting cells, and the area of the outgrowth was not significantly decreased by the treatment until some 48 hours after exposure (Fig. 21).

FIG. 21.—Difference in Area Between Control and Test Cultures.

48 hours after exposure to a $10^{-6}$ g./ml. solution of R 128 in Tyrode for 30 minutes. (Average of five pairs of cultures.)

| | Explant. | Thick outgrowth. | Thin outgrowth. | Total outgrowth. |
|---|---|---|---|---|
| Total C.-T.= | . —0·17 mm.$^2$ | . 6·18 mm.$^2$ | . 2·77 mm.$^2$ | . 8·95 mm.$^2$ |
| Mean C.-T.= | . —0·03 ,, | . 1·24 ,, | . 0·55 ,, | . 1·79 ,, |

After 48 hours' growth without subcultivation, total mitotic counts were found impracticable because of abundant cell overlapping, especially in the control cultures. At this stage the treated cultures showed no sign of recovery, but on the contrary there was a visible difference in the area of the outgrowth as compared with the controls. This was measured with the aid of a camera lucida as shown in Fig. 21.

The statistical analysis of the original figures was done in collaboration with Dr. D. V. Lindley using test " t " for P = 0·05, the $\chi^2$ analysis of contingency tables and the analysis of the variance on the square root of the counts. The effect of treatment on the total number of mitoses was significant in Experiments 1, 2 and 3 as well as the decrease in area in Experiment 4 but there was no quantitative residual effect after transferring the explants to a fresh medium in Experiment 5. The effect on the occurrence of each phase of mitosis was not significant except for a small increase in pro- and metaphase in Experiments 3 and 4.

The more frequent types of abnormalities caused by treatment with low concentrations of the mustard were clumping and scattering of chromatin in pro- and metaphase (Fig. 7, 8, 9), but ana- and telophase abnormalities were also found (Fig. 11 to 16), notably after 48 hours. The percentage of abnormal pro- and metaphases taken together was approximately 35 per cent after 24 hours and 20 per cent after 48 hours, while the number of abnormal ana- and telophases was only 4 per cent after 24 hours and reached 12 per cent after 48 hours.

In spite of the normal appearance of the resting cells, all the evidence obtained from the counts points to the fact that almost 100 per cent of the inhibitory action was exerted during interphase. The deviation in the proportions of pro- and metaphases found in some experiments accounts for less than 3 per cent and 1·5 per cent of all mitoses respectively. The phase distribution would still have remained unaltered, of course, if all stages had been retarded in exactly the same proportion, but the film analysis showed that this was not the case.

*Low power phase-contrast cinemicrography.*

The number of dividing cells per hour was counted throughout the duration of the experiment—12 to 24 hours. This was averaged and expressed as a percentage of the total number of cells present in the first frame (Table III).

TABLE III.

| Concentration of R " 128 " to which the culture had been exposed. | Percentage of dividing cells per hour. |
|---|---|
| None (controls) | 8·75 |
| $10^{-6}$ g./ml. | 3·5 |
| $10^{-5}$ ,, | 0·5 |
| $2 \times 10^{-5}$ g./ml. | 0·4 |
| $0·5 \times 10^{-4}$ ,, | 0·05 |
| $2 \times 10^{-4}$ ,, | 0·00 |

Growth was partly inhibited by very low concentrations of nitrogen mustard ($10^{-6}$ g./ml.), and was almost arrested with higher concentrations ($2 \times 10^{-4}$ and $5 \times 10^{-5}$ g./ml.), when the cultures died within the first 12 hours. The toxic effect of such high doses is shown in Fig. 17*b*.

A good approximation of the duration of one individual mitosis can be obtained by the analysis of low power films. The chromosomes not being resolved, judgment of phase boundaries rests mainly on changes of shape and refringence (Fig. 18).

The average times for the stages of normal mitoses under these conditions were :

Pro + Metaphase, 20 minutes.
Anaphase, 5 minutes.
Telophase, 12 minutes.

No difference was found between the duration of 10 normal mitoses photographed during the first 5 hours and the last 5 hours of an experiment, nor between peripheral and central cells. Thus the possibility of delay in division being due to ageing or to cell crowding was excluded.

These phase-times were not significantly altered in the cultures treated with concentrations of $10^{-6}$ g./ml., although occasionally a cell might remain rounded up for more than double the normal time. With higher concentrations—$10^{-5}$ and $2 \times 10^{-5}$ g./ml.—the proportion of delayed divisions was increased, and in one film reached up to 20 per cent of the recorded instances. Delay was mainly confined to the " rounding-up " period, which was sometimes four times as long as the normal duration (80 instead of 20 minutes). Some cells, however, remained rounded up indefinitely, without showing any signs of anaphase movements. They probably represented the arrested metaphases observed in fixed and stained preparations and in high power phase-contrast films. In one culture treated with a $10^{-5}$ g./ml. solution a cell remained rounded up for 60 minutes, then elongated and continued thus for another 45 minutes, when it again rounded up. A similar case was recorded under high magnification (see below).

Six cells could be followed from one division to another in the control cultures. The intermitotic period varied from 5 to 20 hours, with an average of 10·6 hours. Cells in the middle had a longer resting stage than cells in the periphery of the outgrowth.

In the treated cultures no cell could be found to divide twice in one film (20 to 24 hours), but due to the scarcity of cell divisions it was not possible to decide whether the resting stage was prolonged or whether cells were prevented from dividing again.

*High power phase-contrast cinemicrography.*

The results have been reported elsewhere by Hughes (1950), and I shall only briefly summarize them here. As might have been expected from the observations reported in previous chapters, many of the individual cells examined behaved normally even in relatively high concentrations of the agent (Experiment 10, Table IV). Of 13 individual sequences recorded in cultures treated with concentrations ranging from $2 \times 10^{-5}$ to $10^{-6}$ g./ml., 7 showed some signs of abnormality ; in 3 cleavage was imperfect, distorted or delayed, and in 4 metaphase was retarded or completely inhibited. One cell in which recording started when metaphase was well advanced (Experiment 11, Table IV) remained in metaphase with active bubbling for another 20 minutes and then the nucleus reconstructed without cell division (Fig. 19*a* and *b*). Another remained in metaphase throughout the duration of the film—1 hour 7 minutes 30 seconds—with random chromosome movements and very active bubbling, but no signs of spindle-formation. Other unrecorded instances of arrested metaphases were observed in cultures treated with concentrations of $10^{-5}$ g./ml. or higher. In every experiment the subsequent survival of the cultures was controlled by keeping other cultures identically treated in the incubator for 24 to 48 hours. Concentrations which killed the cultures were not used in these experiments.

It may be concluded, therefore, that under the conditions of the experiment many dividing cells escaped the immediate effect of the poison, and that when an effect was produced it usually appeared in metaphase and occasionally during cleavage.

TABLE IV.—*Some Sequences obtained with High Power Phase-contrast Cinemicrography.*

Expt. No. 10: Prophase to normal cleavage. Dilution: $2 \times 10^{-5}$ g./ml.

| Stage. | Frame No. | Time interval min. | Time interval sec. |
|---|---|---|---|
| Early prophase | 0 | 16 | 24 |
| Onset of metaphase | 408 | 11 | 24 |
| Onset of anaphase | 692 | 5 | 12 |
| Cleavage starts | 722 | 2 | 18 |
| End of cleavage | 780 | 10 | 6 |
| Nuclei are reconstructed | 1055 | | |

Expt. No. 13: Metaphase to normal cleavage. Dilution: $10^{-5}$.

| Stage. | Frame No. | Time interval min. | Time interval sec. |
|---|---|---|---|
| Onset of metaphase | 0 | 9 | 18 |
| Onset of anaphase | 139 | 3 | 54 |
| Cleavage starts | 197 | 4 | 42 |
| End of cleavage | 268 | 10 | 0 |
| Nuclei are reconstructed | 418 | | |

Expt. No. 5: Metaphase to delayed cleavage with reunion of daughter cells. Dilution: $2 \times 10^{-6}$ g./ml.

| Stage. | Frame No. | Time interval min. | Time interval sec. | Notes. |
|---|---|---|---|---|
| Late metaphase | 0 | 2 | 48 | — |
| Onset of anaphase | 29 | 6 | 12 | — |
| Cleavage starts | 99 | 13 | 6 | Incomplete cleavage; the daughter cells remain attached by a bridge of cytoplasm and then fuse again. |
| Nuclei are reconstructed | 247 | 17 | 30 | Mitochondria flowing from one pole to the other of the binucleate cell. |
| End of film | 445 | — | — | Cells still united. Two adult nuclei. |

Expt. No. 11: Metaphase reconstruction (Fig. 19, *a* and *b*). Dilution: $2 \times 10^{-5}$ g./ml.

| Stage. | Frame No. | Time interval min. | Time interval sec. | Notes. |
|---|---|---|---|---|
| Late metaphase | 0 | 3 | 36 | Cell remarkably round. |
| Clumping | 52 | 6 | 30 | Chromosomes clump into small spherical mass. |
| Onset of anaphase ? | 147 | 9 | 6 | There is some attempt at anaphase but the chromosomes move centrally again. |
| Reconstruction starts | 280 | 6 | 42 | Chromosome contours are lost; the nucleus starts to reconstruct without cleavage. |
| Nucleoli appear | 378 | 51 | 30 | There is great increase in size of nucleus and cytoplasm, accompanied by intense bubbling. No cytokinesis ensues and the result is a single mononucleated giant cell, probably a tetraploid. |
| End of film | 1129 | — | — | |

### DISCUSSION.

The effect of R 128 in reducing the number of mitoses under the experimental conditions previously defined is very significant even with small concentrations. From the analysis of the effect of low concentrations it may be inferred that (*a*) this effect is exerted mainly during interphase; (*b*) the action upon dividing cells is on the whole negligible ; when it exists, it appears chiefly in pro- and metaphase during the first 24 hours and begins to be significant in anaphase after this period ; (*c*) the interphase effect is not evidenced by morphological changes in the resting cells, while the mitotic effect is often, though not invariably, accompanied by cytological abnormalities ; and (*d*) the residual effect on growth is quantitatively not significant.

Lasnitzki (1940) found inhibition and retardation of prophase during the first 24 hours after exposure to small doses of X rays, in close similarity to what was found in some of our experiments. This immediate effect, as well as the metaphase abnormalities seen in fixed preparations and in the film recordings made shortly after the addition of the agent, are unspecific since they are also produced by mustard gas (Hughes and Fell, 1949) and by other " spindle inhibitors " (Hughes, 1950). Arrest of mitosis in interphase by nitrogen mustards has also been reported by Bodenstein (1947) in the embryonic ectoderm. The pycnosis and vacuolation of resting cells do not warrant discussion here since they were obtained with concentrations near or above the lethal. Similar toxic effects on chick fibroblasts were described with an aliphatic mustard by Fell and Allsopp (1949) in a wartime report.

The relative increase of anaphase abnormalities during the second 24 hours is of interest in connection with recent work by Loveless and Revell (1949), who found a significant increase in the rate of chromosome breaks and reunion in Vicia after a latent period following exposure to both aliphatic and aromatic nitrogen mustards, including R 128. Unfortunately the resolution obtainable with chick material is not sufficient to discriminate between chromosome fragments and small laggards (Fig. 14).

---

EXPLANATION OF PLATES.

FIGS. 1–16.—Material fixed with Maximow's fluid and stained in Ehrlich's haematoxiylin after hydrolysis in N HCl.

FIG. 17*a*–20*c*.—Phase contrast cine-camera photos of unstained living cells.

FIG. 1–6.—A normal mitotic sequence to show that cell division can proceed normally in the presence of low concentrations of R 128 ($2 \times 10^{-6}$ and $10^{-6}$ g./ml.). $\times$ 1300.

FIG. 7.—Shattering of chromosome material ($10^{-5}$ g./ml.). $\times$ 1300.

FIG. 8.—Clumped chromosomes in late prophase or early metaphase ($2 \times 10^{-6}$ g./ml.). $\times$ 1300.

FIG. 9*a*.—Metaphase ; fusion of nuclear material ($10^{-5}$ g./ml.). $\times$ 900.

FIG. 9*b*.—Metaphase ; clumping of chromosomes and spindle inhibition. Compare with phase-contrast picture of living cell (Fig. 19*a*) ($10^{-6}$ g./ml.). $\times$ 1300.

FIG. 10.—Metaphase ; failure of congression on the plate ($2 \times 10^{-6}$ g./ml.). $\times$ 900.

FIG. 11, 12.—" Stickiness " in early and late anaphase ($10^{-6}$ g./ml.). $\times$ 1300.

FIG. 13, 14.—Lagging chromosomes in early and late anaphase ($10^{-6}$ g./ml.). $\times$ 900 and $\times$ 1300.

FIG. 15, 16.—Formation of a micronucleus in early and late telophase ($10^{-6}$ g./ml.). $\times$ 900.

FIG. 17*a*.—Periphery of a living culture immediately after treatment with a concentrated solution ($10^{-4}$ g./ml.) for 15 minutes. Cells are still unaffected. Approx. $\times$ 120.

FIG. 17*b*.—The same, 12 hours later. All cells are severely damaged. Approx. $\times$ 120.

FIG. 18.—Edge of a control culture showing two metaphases (M), two anaphases (A) and a telophase (T). Approx. $\times$ 120.

FIG. 19*a*.—Spindle inhibition in a living cell 107 minutes after treatment ($2 \times 10^{-6}$ g./ml.). (For description see Table IV, Experiment 11.) Approx. $\times$ 1400.

FIG. 19*b*.—The same cell, 20 minutes later ; metaphase reconstruction without cleavage. Approx. $\times$ 1400.

FIG. 20*a*, *b*, *c*.—Normal anaphase movements in an untreated living cell for comparison. Approx. $\times$ 1400.

Figs. 19*a* and 19*b* are reproduced by kind permission of the *Quart. J. Microsc. Sci.* from the original pictures obtained by A. F. W. Hughes (1950).

1
2
3
4
5
6
7
9a
13
8
9b
11
10
12
14
15
16
17a
17b
A
T
M
A
M
18
19a
19b
20a
20b
20c

Rinaldini.

Two types of effects have been observed in the present investigation, one functional, i.e., retardation of mitosis, and one structural, i.e., chromosome abnormalities, the former suggesting a disturbance of an enzymatic process (Boyland, 1950 ; Barron, Bartlett and Miller, 1948*a* ; Barron *et al.*, 1948*b*), the latter being perhaps more readily explained by direct linkage of the mustard with a structural protein or nucleoprotein (Goldacre, Loveless and Ross, 1949 ; Loveless and Revell, 1949 ; Loveless, 1951 ; Butler, Gilbert and Smith, 1950 ; Gilbert, Overend and Webb, 1951). Although it is beyond the scope of this work to discuss the chemical aspects of the problem, it may be pointed out that these two current views on the mode of action of nitrogen mustards need not be mutually exclusive, since several enzymes have now been demonstrated in the nucleus and in the chromosomes and tryptophane-containing proteins have been isolated from chromosome material. One may venture to speculate that if a large part of the protein skeleton of the chromatid or the spindle fibre were contributed by the enzymes which control mitoses, by analogy with the role of adenosinetriphosphatase in the myofibril, then both the functional and the structural effects could respond to the same mechanism, or even, perhaps, be exerted on the same protein.

### SUMMARY.

The action of p-amino-N-N-di (2-chloroethyl) aniline in concentrations of $2 \times 10^{-4}$ to $10^{-6}$ g./ml. on the growth of tissue cultures was studied by mitotic counts and phase-contrast cinemicrography.

In every experiment a marked decrease of dividing cells was observed. High concentrations kill the cultures and produce gross cytological abnormalities in both dividing and resting cells. With dilute solutions a large proportion of cells are prevented from entering mitosis, but no pycnosis or vacuolation appears. This effect is exerted mainly during interphase. Pro- and metaphase abnormalities are conspicuous and ana- and telophase abnormalities rare during the first 24 hours, anaphase abnormalities increasing after this period. The duration of the first two phases is significantly prolonged in some cells. Cultures recover from these effects when transplanted into a fresh medium after 48 hours and hardly any residual effect can be detected. The possible implications of these findings are briefly discussed.

I wish to thank Professor J. F. Danielli for having introduced me to the subject, Dr. Honor B. Fell for her encouragement and valuable advice, Dr. A. F. W. Hughes for his help and guidance in the cinemicrography techniques, Dr. D. V. Lindley for his collaboration in the statistical analysis, and the technical staff of the Strangeways Laboratory, especially Mr. L. J. King, for their ready and efficient assistance.

I am indebted to the British Council for a grant that made this work possible.

### REFERENCES.

AUERBACH, C., AND ROBSON, J. M.—(1947) *Proc. Roy. Soc. Edinb.*, **62**, 284.

BARRON, E. S. G., BARTLETT, G. R., AND MILLER, Z. B.—(1948*a*). *J. exper. Med.*, **87**, 489.

*Iidem*, MEYER, J., AND SEEGMILLER, J. E.—(1948*b*) *Ibid.*, **87**, 503.

BODENSTEIN, D.—(1947) *J. exp. Zool.*, **104**, 311.

Boyland, E.—(1950) *Biochem. et Biophys. Acta*, **4**, 293.
*Idem* and Horning, E. S.—(1949) *Brit. J. Cancer*, **3**, 118.
Butler, J. A. V., Gilbert, L. A., and Smith, K. A.—(1950) *Nature*, **165**, 714.
Everett, J. L., and Ross, W. C. J.—(1949) *J. chem. Soc.*, 1972.
Fell, H. B., and Allsopp, C. B.—(1949) *Cancer Res.*, **9**, 238.
Gilbert, L. M., Overend, W. G., and Webb, M.—(1951) *Exp. Cell Res.*, **2**, 349.
Goldacre, R. J., Loveless, A., and Ross, W. C. J.—(1949) *Nature*, **163**, 667.
Haddow, A.—(1947) *Brit. med. Bull.*, **4**, 417.
*Idem*, Kon, C. A. R., and Ross, W. C. J.—(1948) *Nature*, **162**, 824.
Hanby, W. E., Bartley, G. S., Powell, E. O., and Rydon, H. N.—(1947) *J. chem. Soc.*, 519.
Hughes, A. F. W.—(1950) *Quart. J. microsc. Sci.*, **91**, 251.
*Idem* and Fell, H. B.—(1949) *Ibid.*, **90**, 37.
*Idem* and Swann, M. M.—(1948) *J. exp. Biol.*, **25**, 45.
Lasnitzki, I.—(1940) *Brit. J. Radiol.*, **13**, 279.
Loveless, A.—(1951) *Nature*, **167**, 338.
*Idem* and Revell, S. H.—(1949) *Ibid.*, **164**, 938.
Ross, W. C. J.—(1949) *J. chem. Soc.*, 183.
Tatum, E. L.—(1947) *Ann. N.Y. Acad. Sci.*, **49**, 87.